# Whole Cell-SELEX Aptamers for Highly Specific Fluorescence Molecular Imaging of Carcinomas *In Vivo*

**DOI:** 10.1371/journal.pone.0070476

**Published:** 2013-08-12

**Authors:** Hui Shi, Wensi Cui, Xiaoxiao He, Qiuping Guo, Kemin Wang, Xiaosheng Ye, Jinlu Tang

**Affiliations:** 1 State Key Laboratory of Chemo/Biosensing and Chemometrics, Hunan University, Changsha, China; 2 College of Chemistry and Chemical Engineering, Hunan University, Changsha, China; 3 Institute of Biology, Hunan University, Changsha, China; 4 Key Laboratory for Bio-Nanotechnology and Molecule Engineering of Hunan Province, Changsha, China; The University of Hong Kong, Hong Kong

## Abstract

**Background:**

Carcinomas make up the majority of cancers. Their accurate and specific diagnoses are of great significance for the improvement of patients' curability.

**Methodology/Principal Findings:**

In this paper, we report an effectual example of the *in vivo* fluorescence molecular imaging of carcinomas with extremely high specificity based on whole cell-SELEX aptamers. Firstly, S6, an aptamer against A549 lung carcinoma cells, was adopted and labeled with Cy5 to serve as a molecular imaging probe. Flow cytometry assays revealed that Cy5-S6 could not only specifically label *in vitro* cultured A549 cells in buffer, but also successfully achieve the detection of *ex vivo* cultured target cells in serum. When applied to *in vivo* imaging, Cy5-S6 was demonstrated to possess high specificity in identifying A549 carcinoma through a systematic comparison investigation. Particularly, after Cy5-S6 was intravenously injected into nude mice which were simultaneously grafted with A549 lung carcinoma and Tca8113 tongue carcinoma, a much longer retention time of Cy5-S6 in A549 tumor was observed and a clear targeted cancer imaging result was presented. On this basis, to further promote the application to imaging other carcinomas, LS2 and ZY8, which are two aptamers selected by our group against Bel-7404 and SMMC-7721 liver carcinoma cells respectively, were tested in a similar way, both *in vitro* and *in vivo*. Results showed that these aptamers were even effective in differentiating liver carcinomas of different subtypes in the same body.

**Conclusions/Significance:**

This work might greatly advance the application of whole cell-SELEX aptamers to carcinomas-related *in vivo* researches.

## Introduction

Carcinomas make up the majority of cancers and pose a great threat to human health. They are cancers of the epithelial cells and begin in the skin or tissues lining or covering body organs like lung, liver, breast, etc [1], [2]. An accurate and specific diagnosis of carcinoma to identify its type or even subtype is especially crucial and directly determines the treatment design from doctors [3], [4]. For example, non-small cell lung cancer and small cell lung cancer both belong to lung carcinomas, but their treatments are greatly different. The former often takes operation method and the latter preferably adopts chemotherapy and radiotherapy. Therefore, the success of highly specific detection of carcinomas is of great significance to the improvement of therapy effects and the curability of patients.

In recent years, as a rapidly emerging field, molecular imaging has received much attention in biomedical research and clinical diagnoses [5], [6]. Different from traditional imaging technologies which are based on morphological information, molecular imaging typically utilizes specific molecular probes to image particular biological events. These molecular imaging probes are designed and fabricated to be able to study molecular-level abnormalities in a non-invasive, real-time and in-situ way, thus affording potentials for realizing *in vivo* specific diagnoses of diseases like cancers [7]. Among the various reported molecular imaging probes, such as antibodies [8], ligands [9], enzyme substrates [10] and so on, nucleic acid aptamers are relatively new and promising, which are single-stranded oligonucleotides with distinct binding properties to diverse targets including cancer markers and cells [11], [12]. Aptamers not only possess a number of inherent advantages like excellent affinity and specificity for target recognition, economical and reproducible synthesis, non-toxicity and flexible modification, but also exhibit rapid blood clearance, fast tumor penetration and favorable target-to-noise ratios at early time points for *in vivo* applications because of their small sizes [13], [14]. Particularly, the recently developed whole cell-SELEX (systematic evolution of ligands by exponential enrichment) technology, a process to select aptamers with whole intact cancer cells being targets [15], has further strengthened the function of aptamers in identifying molecular differences between cancer cells. In that protocol, a counter selection has been introduced using closely related cells as the control, thus producing highly specific aptamers capable of differentiating cancer cells even in the same type.

Up to now, a variety of aptamers which are against different cancer cells respectively, including leukemia [16], lymphoma [17], lung cancer [18], [19], ovarian cancer [20], and colorectal cancer [21], etc., have been selected through whole-cell SELEX. Although most of them were successfully applied to *in vitro* cancer diagnoses and related researches [22], [23], [24], there are only a few aptamers have been tested in real biological samples, especially for *in vivo* imaging [25], [26], [27]. Our group, for the first time, have demonstrated that near-infrared dye Cy5-labeled TD05 aptamer could effectively recognize Ramos lymphoma cells in mice and achieve fluorescence cancer imaging with high specificity [26]. On this basis, an activatable aptamer probe based on target-triggered conformation alteration was further designed using Sgc8c aptamer of CCRF-CEM leukemia cells, and displayed the contrast-enhanced *in vivo* cancer imaging [27]. These studies substantially support the feasibility of aptamers as specific molecular imaging probes, but most of them are only concentrated on blood cancer researches. As for carcinomas, which are relatively more representative and important types of cancers, their *in vivo* fluorescence imaging based on whole cell-SELEX aptamers have not yet been reported.

In the present paper, human lung cancer, a leading cause of cancer mortality worldwide [28], was chosen as the carcinoma model for *in vivo* imaging study. The corresponding aptamer S6 which is evolved from whole cell-SELEX and is against A549 adenocarcinoma cells [19], was adopted and labeled with Cy5 to serve as a fluorescence imaging probe. A systematic study, including *in vitro* flow cytometry assay, *in vivo* fluorescence imaging and *ex vivo* organ imaging validation, was then carried out to demonstrate its specific recognition ability to differentiate different cancer types. Subsequently, to further testify aptamers' capability of imaging other carcinomas, we utilized aptamers selected against hepatocellular carcinoma cells by whole-cell SELEX in our lab to achieve the identification of different cancer cells in the same type both *in vitro* and *in vivo*.

## Materials and Methods

### Ethics Statement

This study was carried out in strict accordance with the Regulations for the Management of Laboratory Animals of the Ministry of Science and Technology of the People's Republic of China. The protocol was approved by the Committee on the Ethics of Animal Experiments of Hunan Provincial Laboratory Animal Center [Permit Number: SYXK (Xiang) 2008–0001]. All surgery was performed under sodium pentobarbital anesthesia, and all efforts were made to minimize suffering.

### Chemicals and materials

All the DNA probes reported in this article were custom-designed and then synthesized by Sangon Biotech. (Shanghai, China) Co., Ltd. Sequences of the oligos are listed in **Table 1**. Dulbecco's phosphate buffered saline was purchased from Sigma. Mouse serum was obtained from Dingguo Changsheng Biotechnology Co., Ltd. All other reagents were of the highest grade available. Deionized water was obtained through the Nanopure Infinity^TM^ ultrapure water system (Barnstead/Thermolyne Corp.). Binding buffer was prepared by adding 1 mg/mL BSA and 10% fetal bovine serum into the Dulbecco's PBS containing 4.5 g/L glucose and 5 mM MgCl_2_. Washing buffer was prepared by adding 0.1% NaN_3_ into the Dulbecco's PBS containing 4.5 g/L glucose and 5 mM MgCl_2_.

**Table 1:** List of the DNA sequences used in the experiments.

Aptamer	Sequence
Cy5-labeled S6	5′-Cy5-GTG GCC AGT CAC TCA ATT GGG TGT AGG GGT GGG GAT TGT GGG TTG-3′
Cy5-labeled library	5′-Cy5-(NNN)_15_-3′
Cy5-labeled LS2	5′-Cy5-ATG AGA GCG TCG GTG TGG TAA TGG AAT GTG GGA GGG GGA CTC AGG ACA GTC ACG GlesGA CAT GTA GGA GGG TGC GGA AGT A-3′
Cy5-labeled ZY8	5′-Cy5-TTG ACT TGC CAC TGA CTA CCA CCT TTC TAG GTG GTT GAG CTG AAG ATC GTA CCG TGA AGT CAG TCG GTC GTC ATC-3′

### Cells

Seven *in vitro* cultured cell lines were used in this study: A549 (human lung carcinoma), Hela (human cervical carcinoma), MCF-7 (human breast carcinoma), Tca8113 (human tongue carcinoma), Bel-7404 (human hepatocellular carcinoma), SMMC-7721 (human hepatocellular carcinoma), and L02 (normal human hepatocytes). Thereinto, A549, Hela and MCF-7 were obtained from American Type Culture Collection (ATCC). Tca8113 was purchased from the China Center for Type Culture Collection (Wuhan University). Bel-7404, SMMC-7721 and L02 were purchased from the Shanghai Institute of Cell Biology of the Chinese Academy of Science. All cell lines were cultured in RPMI 1640 medium supplemented with 10% fetal calf serum, 100 μg/mL streptomycin and 100 IU/mL penicillin. Cells were all incubated at 37°C in a humidified incubator containing 5% CO_2_.

### Tumor growth

Male athymic BALB/c (Balb/C-nu) mice were purchased from the Shanghai SLAC Laboratory Animal Co., Ltd. (BALB/c). Four- to six-week-old nude mice received a subcutaneous injection of 5×10^6^ *in vitro*-propagated human cancer cells into the backside. Tumors were then allowed to grow to 0.5–1.5 cm in diameter for 20–40 days.

### *Ex vivo* culture of cancer cells

*Ex vivo* cultured cancer cells were derived from the primary tumor tissue explants through procedures described as follows. Under a sterile condition, tumor tissues were isolated from the mice and washed for three times with D-hank's balanced salt solution supplemented with penicillin/streptomycin. Then, tumor tissues were placed in dishes containing cell culture medium and cut into slices as small as possible to ensure enough cancer cells were dispersed in the medium. After the tissue fragments were removed from the medium, *ex vivo* cancer cells were cultured in RPMI 1640 medium supplemented with 20% fetal calf serum, 100 μg/mL streptomycin and 100 IU/mL penicillin (37°C, 5% CO_2_).

### Flow cytometry assays

Anchorage-dependent cells were firstly harvested with 0.02% EDTA and/or 0.5% trypsin to prepare cell suspensions for the following flow cytometry assays. Then, Cy5-labeled DNA probes were incubated with 2×10^5^ cells in 200 µL binding buffer or mouse serum at 4°C for 1 h in the dark. Next, cells were washed twice with 0.5 mL of washing buffer and resuspended in 0.3 mL of binding buffer. The fluorescence was determined with a FACScan cytometer (BD Biosciences, Mountain View, CA, USA) by counting 10, 000 events.

### *In vivo* fluorescence imaging

BALB/c nude mice, with or without tumors, were anesthetized with both tranquilizer and anesthetic. Once the mice were anesthetized motionlessly, a 200 μL volume of physiological saline containing 0.5 nmol of Cy5-labeled DNA probes and 5 nmol of unlabeled random oligonucleotide was injected intravenously via the tail vein. At certain time points, fluorescence images of live mice were taken by an IVIS Lumina II *in vivo* imaging system (Caliper LifeSicence, USA). A 640 nm (±15 nm) bandpass filter and a 695–770 nm bandpass filter were selected as the excitation filter and the emission filter respectively.

### *Ex vivo* organ imaging

After *in vivo* imaging, the mice injected with different Cy5-labeled DNA probes were killed by cervical dislocation under narcosis at 3 h postinjection. The anatomized mice and dissected organs, including liver, kidney, spleen, lung, heart, spermary, bladder, brain, intestine and tumor tissue, were imaged with the IVIS Lumina II *in vivo* imaging system as described above.

## Results and Discussion

### Specific detection of lung carcinoma cells *in vitro*

**Figure 1:**
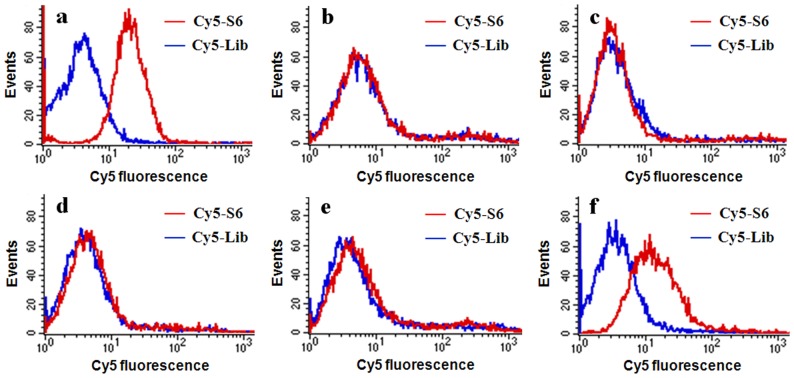
*In vitro* recognition specificity investigation of Cy5-S6. Flow cytometry assays of different *in vitro* cultured cells in binding buffer using Cy5-S6, including (a) A549, (b) L02, (c) Tca8113, (d) MCF-7, and (e) Hela cells. (f) Identification of *ex vivo* cultured A549 cells in mouse serum based on Cy5-S6. Cy5-Lib was used as the negative control probe.

To demonstrate the feasibility of aptamers selected by whole cell-SELEX in specifically detecting target cells, flow cytometry assays were conducted by incubating Cy5-labeled S6 (Cy5-S6) with different cells in binding buffer, including normal hepatocyte L02 cells, lung carcinoma A549 cells, tongue carcinoma Tca8113 cells, breast carcinoma MCF-7 cells and cervical carcinoma Hela cells. Cy5-labeled 45mer sequence-randomized DNA library (Cy5-Lib) was used as the negative control probe. As shown in **Figure 1a**, compared with nonspecific signals from Cy5-Lib, the specific aptamer Cy5-S6 exhibited much higher fluorescence labeling in *in vitro* cultured A549 cells. In contrast, little signal differences between Cy5-S6 and Cy5-Lib stained cells were detected in other four cell lines, no matter normal cells or carcinoma cells (**Figure 1b–e**). It was obvious that the Cy5-S6 aptamer probe could afford substantial specific affinity against *in vitro* cultured A549 lung carcinoma cells. Thereupon, in order to further investigate its applicability to circumstance changes, *ex vivo* cultured cells taken from tumor tissues of A549 tumor-bearing mice were prepared and incubated with Cy5-S6 in mouse serum. Results in **Figure 1f** clearly revealed that Cy5-S6 could effectively retain its affinity to detect target cells even in complex biofluids, which undoubtedly paved the way for its *in vivo* application.

### Specific fluorescence imaging of lung carcinoma *in vivo*

Before the implementation of aptamer-based cancer imaging in mice, a primary study on the biological distribution and metabolic behaviors of Cy5-S6 was carried out. After being injected into nude mice without tumors via tail vein, the temporal distribution of Cy5-S6 was monitored and imaged *in vivo* from both backside and abdomen (**Figure S1A**). Cy5-S6 was observed to circulate throughout the animal within 5 min, and much stronger fluorescence signals could be seen in kidney and liver. With the postinjection time passing by, fluorescence in the whole body faded little by little and bright signals gradually shifted to the intestine and urinary bladder. It was hypothesized that Cy5-S6 might be excreted from the animal's body through the enteron and renal routes, which was further confirmed by the *ex vivo* organ imaging results (**Figure S1B**).

**Figure 2:**
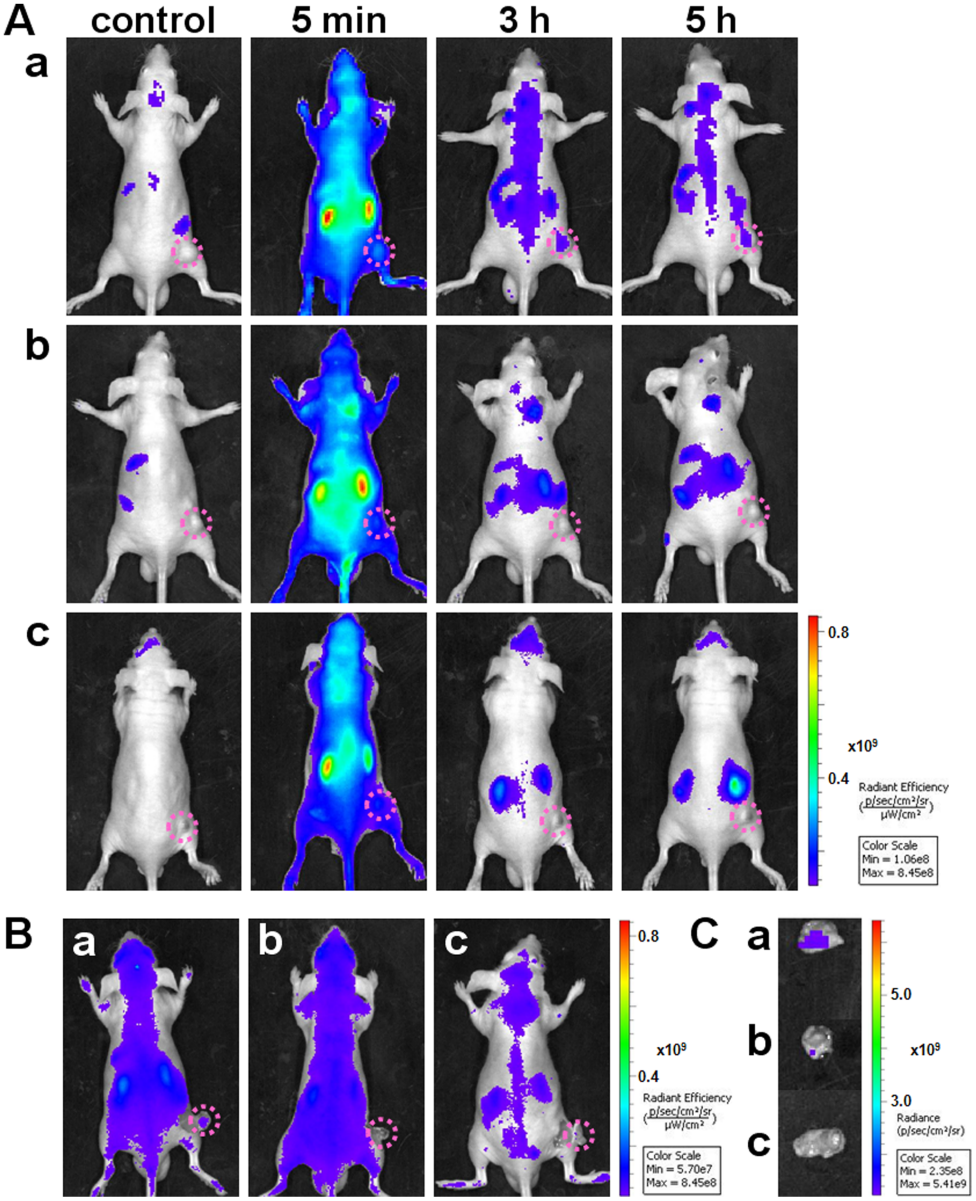
*In vivo* imaging specificity investigation of Cy5-S6. Tumor-bearing mice were intravenously injected with different Cy5-labeled DNA probes and then imaged. (A) Time-lapse *in vivo* fluorescence imaging. (B) Images at 3 h postinjection with tumor tissues uncovered. (C) Images of the isolated tumor tissues. (a) A549 tumor-bearing mice injected with Cy5-S6 or (b) Cy5-Lib, (c) Tca8113 tumor-bearing mice injected with Cy5-S6. The pink circle in every image locates the tumor site.

Subsequently, a systematic comparison investigation was performed to validate the capability of Cy5-S6 in specific fluorescence imaging of A549 lung carcinoma *in vivo* by using Cy5-Lib and the Tca8113 tongue carcinoma as the control probe and tumor model respectively (**Figure 2A**). Without regard to the interference of metabolic organs, the tumor site could be roughly identified with a brighter fluorescence just after the injection of Cy5-S6 into A549 tumor-bearing mouse for 5 min (**group a**). Due to the time-dependent clearance of Cy5-S6 in non-target areas, the tumor imaging contrast was greatly enhanced at 3 h postinjection time, and until 5 h, the clear tumor figure could still be imaged. In contrast, for A549 tumor-bearing nude mice injected with Cy5-Lib, favorable tumor-to-background ratios were rarely measured from early to late time points (**group b**). It should be the specific sequence that endowed Cy5-S6 with the function to recognize and target A549 lung carcinoma cells *in vivo*, thus slowing down the aptame' s clearance in tumor site and leading to perfect cancer images. Furthermore, the *in vivo* imaging specificity of Cy5-S6 was also tested by using Tca8113 tumor-bearing nude mice as the control carcinoma model (**group c**). It was found that at 3 h postinjection time, fluorescence signals in the Tca8113 tumor were almost cleared, which positively demonstrated that Cy5-S6 could afford the unique ability to specifically distinguish between A549 lung carcinoma and Tca8113 tongue carcinoma *in vivo*. To be specific, although Cy5-S6 could be significantly captured by the target tumor, its nonspecific accumulation in the control tumor was faint. In order to confirm the above non-invasive monitoring results, the tumor tissues were uncovered and then isolated for *ex vivo* imaging validation. As displayed in **Figure 2B and C**, at 3 h postinjection time, the fluorescence emitted from the A549 tumor with Cy5-S6 was obviously brighter than those from the A549 tumor with Cy5-Lib and the Tca8113 tumor with Cy5-S6.

### Specific fluorescence imaging of nude mice simultaneously grafted with different carcinomas

**Figure 3:**
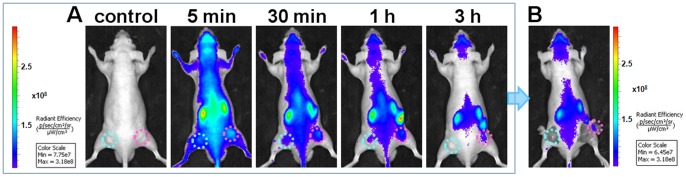
*In vivo* specific fluorescence imaging in the same mouse body. Images were acquired after an intravenous injection of Cy5-S6 in a nude mouse simultaneously bearing an A549 tumor (pink circles) and a Tca8113 tumor (cyan circles). (A) Time-lapse fluorescence imaging. (B) Imaging at 3 h postinjection with the tumor tissues uncovered.

Then we implemented Cy5-S6 for *in vivo* imaging of a nude mouse which was simultaneously grafted with different carcinomas, including a A549 tumor on the right and a Tca8113 tumor on the left. In comparison with the Tca8113 tumor site, a much brighter fluorescence signal could be clearly detected at the A549 tumor site even at very early time points (**Figure 3**). With the extension of postinjection time, fluorescence signals in the whole body including the target and non-target tumor areas faded gradually due to the excretion of Cy5-S6. Still, the potent interaction between aptamer and its target could afford a much longer retention time of Cy5-S6 in target areas, which thus led to a gradual enhancement of A549 tumor-to-background contrast. During the whole imaging process, the signal of A549 tumor was found to be always much stronger than that of the Tca8113 tumor. Especially at 3 h postinjection time, the fluorescence from the left tumor was almost disappeared while a distinct profile was still imaged for the right tumor. This was further testified by uncovering these two tumor tissues, which undoubtedly demonstrated that Cy5-S6 held a great potential to effectively differentiate different cancer types even in the same body. The high specificity of Cy5-S6 indicates the function of aptamers generated by whole cell-SELEX to image particular biological events *in vivo*.

### Application of whole cell-SELEX aptamers for highly specific identification of other carcinomas *in vitro* and *in vivo*

**Figure 4:**
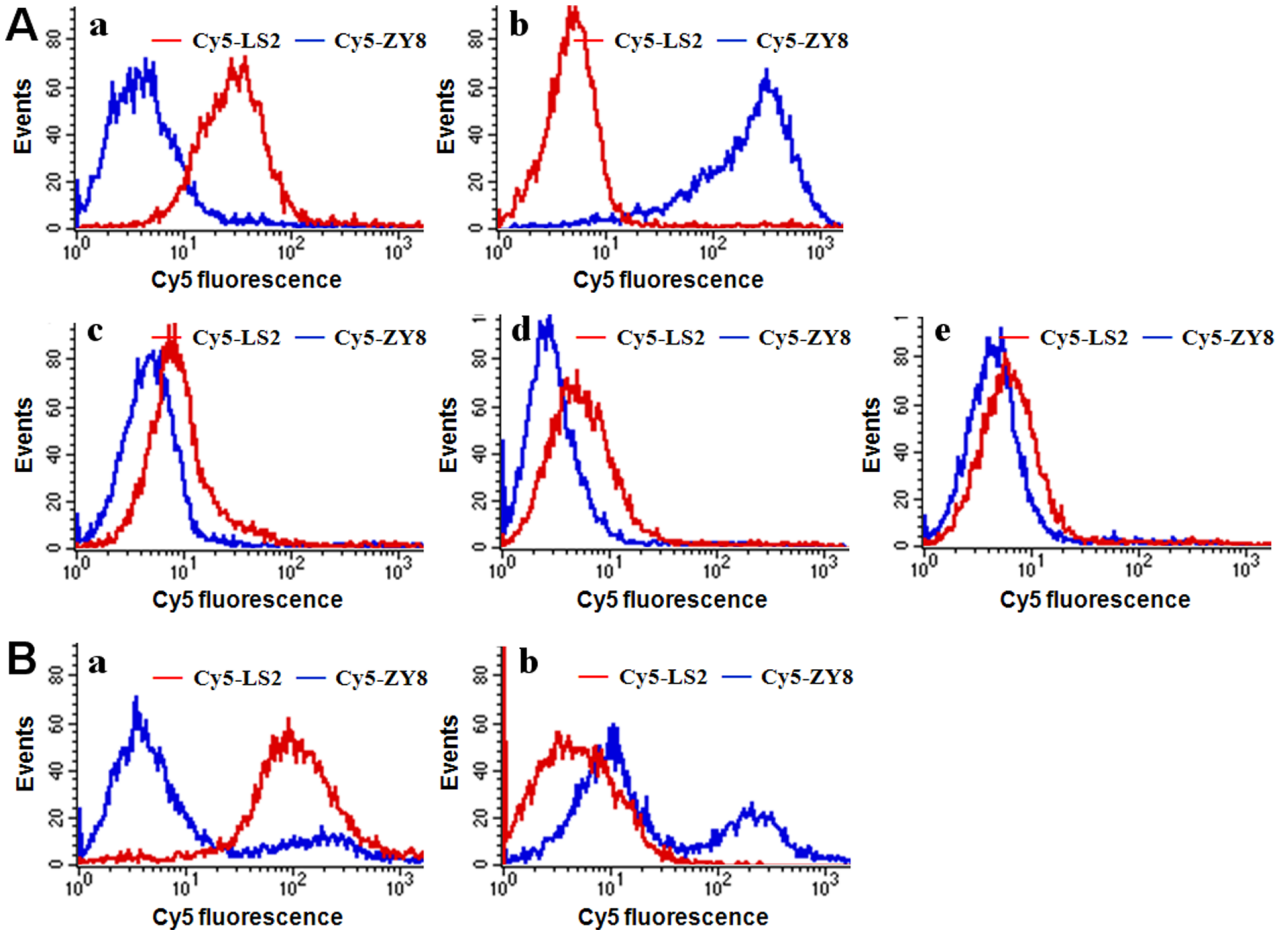
*In vitro* recognition specificity investigation of liver carcinoma aptamers. (A) Flow cytometry assays of different *in vitro* cultured cells in binding buffer using Cy5-labeled aptamers, including (a) Bel-7404, (b) SMMC-7721, (c) L02, (d) MCF-7, and (e) Hela cells. (B) Identification of *ex vivo* cultured (a) Bel-7404 and (b) SMMC-7721 liver carcinoma cells in mouse serum based on Cy5-labeled aptamers.

To promote the application of whole cell-SELEX aptamers in molecular imaging to other carcinomas, hepatocellular cancer was chosen for the *in vitro* and *in vivo* investigations. It is the sixth most prevalent cancer and the third most frequent causes of cancer-related death [29]. Two aptamers, LS2 [30] and ZY8 (unpublished data) that were selected for Bel-7404 and SMMC-7721 liver carcinoma cells respectively through whole cell-SELEX by our group, were adopted as molecular probes. After labeled with Cy5, these two aptamer probes were applied to detect target cancer cells *in vitro* with each other being the control probe (**Figure 4**). Results showed that Cy5-labeled LS2 (Cy5-LS2) could not only specifically recognize *in vitro* cultured Bel-7404 cells in binding buffer, but also effectively achieve the detection of *ex vivo* cultured Bel-7404 cells in mouse serum with much higher fluorescence signals than Cy5-labeled ZY8 (Cy5-ZY8). Moreover, by comparison with control cell lines, including L02, MCF-7, Hela and SMMC-7721, Cy5-LS2 exhibited substantial specificity for the identification of Bel-7404 liver carcinoma cells from other cell types or even subtypes. In like manner, *in vitro* cultured SMMC-7721 cells in binding buffer were also selectively labeled by Cy5-ZY8. However, the staining pattern of *ex vivo* cultured SMMC-7721 cells in mouse serum was somewhat different: presenting two groups of cells with inconsistent fluorescence intensities. It was hypothesized that the complex biological environment during the growth of SMMC-7721 tumors in nude mice might slightly influence the expression of target receptors in *ex vivo* cultured SMMC-7721 cells. Whether or no, these two aptamers both exhibited perfect binding specificity to identify different cancer types and even subtypes *in vitro*.

**Figure 5:**
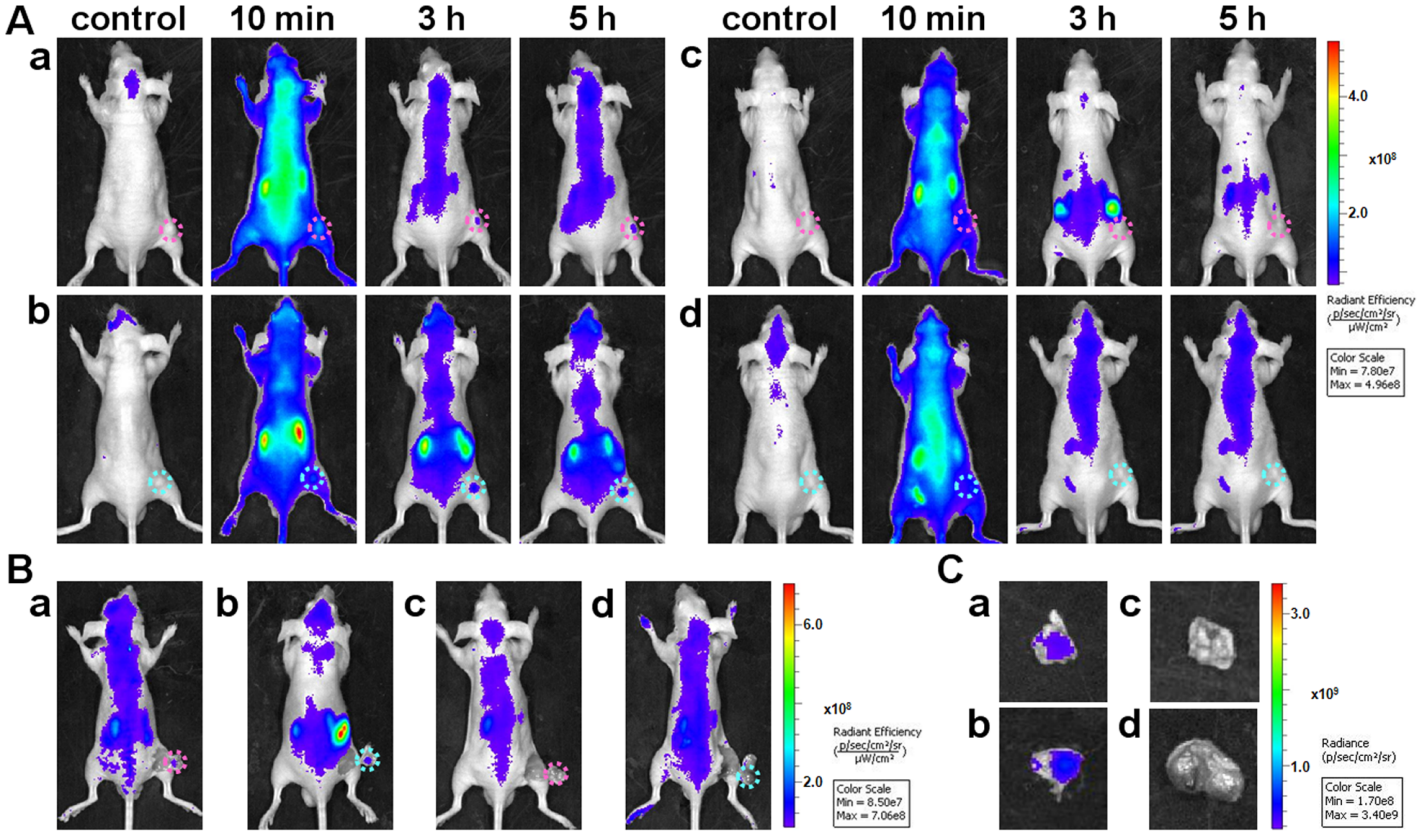
*In vivo* imaging specificity investigation of liver carcinoma aptamers. (A) Time-lapse *in vivo* fluorescence imaging. (B) Images at 3 h postinjection with tumor tissues uncovered. (C) Images of the isolated tumor tissues. Mice grafted with (a, c) Bel-7404 tumors (pink circles) or (b, d) SMMC-7721 tumors (cyan circles) were intravenously injected with (a, b) Cy5-LS2 or (c, d) Cy5-ZY8, and then imaged.

**Figure 6:**
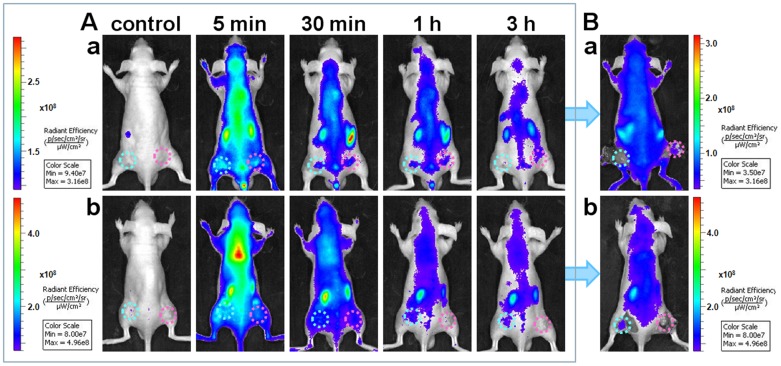
*In vivo* highly specific differentiation between two liver carcinomas in the same mouse body. (A) Time-lapse fluorescence imaging. (B) Imaging at 3 h postinjection with the tumor tissues uncovered. Images were acquired after an intravenous injection of (a) Cy5-LS2 or (b) Cy5-ZY8 in nude mice simultaneously bearing Bel-7404 tumors (pink circles) and SMMC-7721 tumors (cyan circles).

Then, *in vivo* imaging experiments were carried out by intravenously injecting these two Cy5-labeled aptamers into Bel-7404 or SMMC-7721 liver carcinoma-bearing nude mice respectively. These two carcinomas were regarded as the control for each other. Results in **Figure 5A** showed that the metabolism behavior and imaging mechanism of these two Cy5-labeled liver carcinomas aptamers were similar to those of Cy5-S6 in mice bodies. As a result of the specific recognition function, Cy5-LS2 was found to only work in imaging the target Bel-7404 tumor, and Cy5-ZY8 was successfully used to probe the SMMC-7721 tumor site. That was further validated by the subsequent imaging of tumor tissues uncovered (**Figure 5B**) and isolated tumors (**Figure 5C**). The high selectivity between different liver carcinomas was particularly revealed in **Figure 6A**, which illustrates the time-lapse images of nude mice simultaneously grafted with a Bel-7404 tumor on the right and a SMMC-7721 tumor on the left after the injection of different liver carcinoma aptamers. It was clearly observed that the tumor on the right was lightened by Cy5-LS2 and the tumor on the left was indicated by Cy5-ZY8. Moreover, the imaging result of tumor tissues uncovered from above mice further demonstrated that Cy5-LS2 and Cy5-ZY8 perfectly retained their high affinity and specificity for *in vivo* target cells (**Figure 6B**).

## Conclusions

A549 lung cancer cells, Bel-7404 and SMMC-7721 liver cancer cells being used as models, aptamers generated by whole cell-SELEX have been successfully applied as molecular probes for fluorescence imaging of carcinomas in living mice. The systematic investigation positively revealed that whole cell-SELEX aptamers could afford robust recognition capability to target cells both *in vitro* and *in vivo*. In particular, their high specificity for cancer imaging was repetitiously confirmed, which greatly supported their potential application to diagnoses of different cancer types and even subtypes in complex systems. This work not only makes a great contribution to introduce whole cell-SELEX aptamers into carcinomas-related *in vivo* researches, but also once more attests the overall efficacy of the whole cell-SELEX method in generating molecular imaging probes to target specific biological events and to identify disease biomarkers.

## Supporting Information

Figure S1 **Biological distribution investigation of Cy5-S6.** (A) Time-lapse *in vivo* fluorescence imaging of Cy5-S6 in a normal nude mouse without tumors through an intravenous injection. (a) back imaging, (b) abdomen imaging. (B) Image of the organs in a normal nude mouse without tumors after intravenous injection of Cy5-S6 for 3 h. (br = brain; li = liver; ki = kidney; sp = spleen; lu = lung; he = heart; s.i. = small intestine; l.i. = large intestine; st = stomach; spe = spermatophore; u.b. = urinary bladder; in = intestine).
(TIF)
