# Supplementary figures and images for: Whole Cell-SELEX Aptamers for Highly Specific Fluorescence Molecular Imaging of Carcinomas In Vivo
Source: PLoS One. 2013 Aug 12;8(8):e70476. doi: 10.1371/journal.pone.0070476 (PMC3741280; doi:10.1371/journal.pone.0070476)

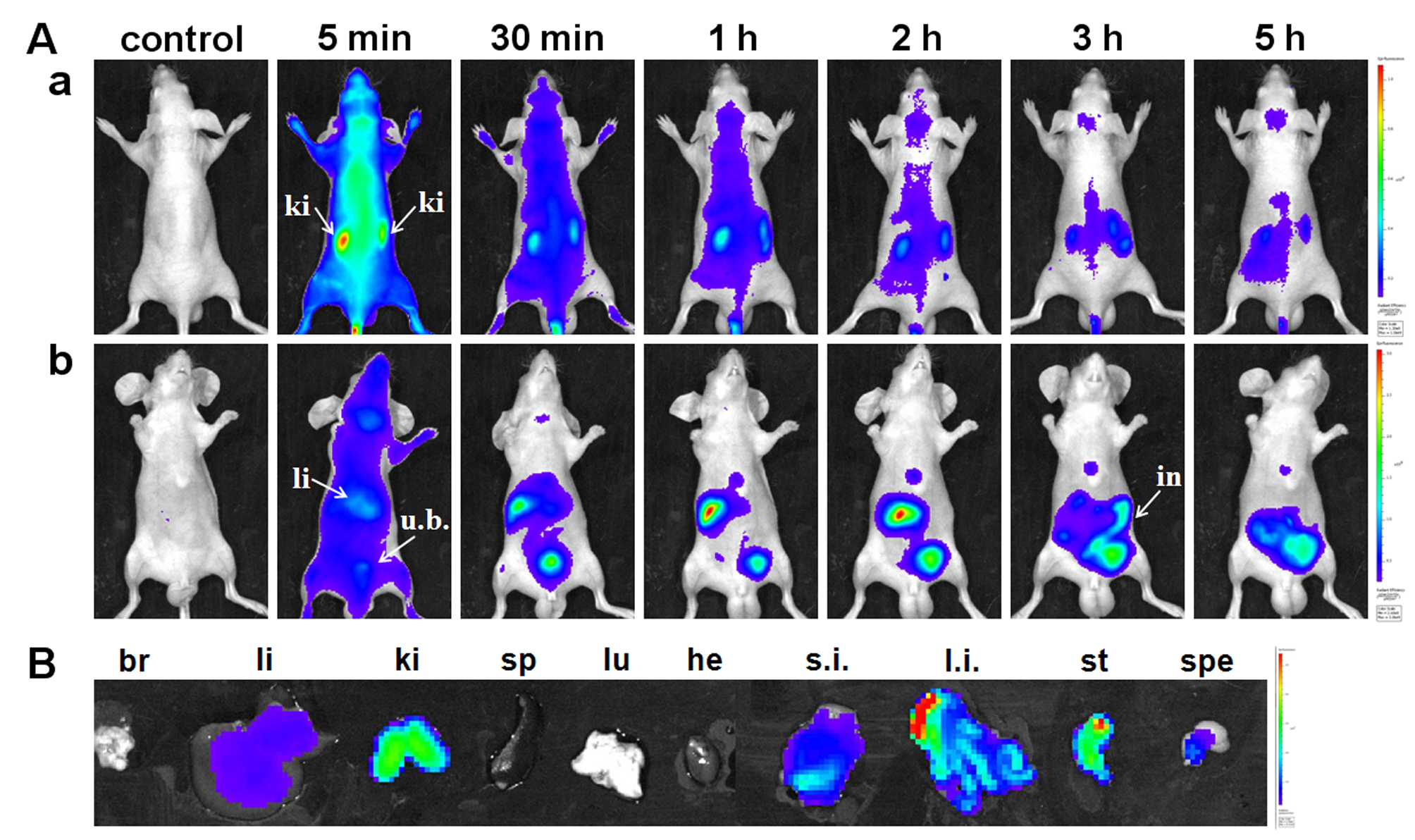

Supplement: Figure S1 — Biological distribution investigation of Cy5-S6. (A) Time-lapse in vivo fluorescence imaging of Cy5-S6 in a normal nude mouse without tumors through an intravenous injection. (a) back imaging, (b) abdomen imaging. (B) Image of the organs in a normal nude mouse without tumors after intravenous injection of Cy5-S6 for 3 h. (br = brain; li = liver; ki = kidney; sp = spleen; lu = lung; he = heart; s.i. = small intestine; l.i. = large intestine; st = stomach; spe = spermatophore; u.b. = urinary bladder; in = intestine). (TIF) [file pone.0070476.s001.tif]
